# Molecular Design of Microcapsule Shells for Visible Light-Triggered Release

**DOI:** 10.3390/polym11050904

**Published:** 2019-05-17

**Authors:** Domenico Pirone, Valentina Marturano, Rita Del Pezzo, Susana Fernández Prieto, Todd Underiner, Marta Giamberini, Bartosz Tylkowski

**Affiliations:** 1 Department of Chemical Engineering, Rovira i Virgili University, Av. Països Catalans 26, 43007 Tarragona, Spain; domenico.pirone@estudiants.urv.cat (D.P.); rita.delpezzo1@estudiants.urv.cat (R.D.P.); marta.giamberini@urv.cat (M.G.); 2 Centre Tecnològic de la Química de Catalunya, Carrer Marcelli Domingo s/n, 43007 Tarragona, Spain; 3 Procter & Gamble Services Company n.v., Temselaan 100, 1853 Strombeek-Bever, Belgium; fernandezprieto.s@pg.com; 4 Department of Chemical, Materials, and Production Engineering (DICMAPI), University of Naples “Federico II”, P. le Tecchio, 80, 80125 Napoli, Italy; valentina.marturano@unina.it; 5 The Procter and Gamble Company, 6210 Center Hill Avenue, Cincinnati, OH 45224, USA; underiner.tl@pg.com

**Keywords:** encapsulation, photo-triggered release, modified azobenzene

## Abstract

The development of photo-responsive capsules to tune and control the sustained-release of encapsulated actives is a fascinating and challenging route to improve the performances and effectiveness of a wide range of delivery applications. In this work, we report the preparation of visible light-responsive capsules obtained via oil-in-water interfacial polycondensation between modified diacyl-chloride azobenzene moiety and diamine flexible spacer in the presence of cross-linkers with different structures and functionalities. The effect on the release profile of the encapsulated perfume oil was investigated using three flexible spacers with different lengths (1,8-diaminooctane; 1,6-diaminohexane and 1,4-diaminobutane) and two types of cross-linkers (1,3,5-benzenetricarbonyl trichloride and melamine). We analyzed how the properties of microcapsules can be tailored changing the design of the shell structure. Fine tuning of the perfume release profiles was obtained. The changes in capsules size and morphology due to visible light irradiation were monitored via light scattering, optical microscopy and atomic force microscopy. Perfume release was 50% faster in the systems prepared with melamine as the cross-linker. Modelling studies were carried out to support the discussion of the experimental results.

## 1. Introduction

Microcapsules (MC) are vastly investigated core-shell systems able to protect, deliver and stabilize many substances, entrapping them in a core part of a well-defined shell. The utility of microcapsules is represented by the possibility of storing their cargo material for a prolonged time or tailoring its release in a targeted way [1]. Microencapsulation has numerous applications in areas such as pharmaceutical, agricultural, medical and food industries, and it is widely used in the encapsulation of essential oils [2], flavorings [3], drugs [4,5] and dyes [6], among others [7,8,9,10,11,12,13,14].

To create a useful delivery system, the most challenging task is to trigger and modulate the release of the encapsulated substances. Light [15], temperature [16] and pH change [17] are only some of the several triggers that have been investigated by scientists during the last decades to boost microcapsule wall morphological changes. Photo-responsive materials for the design of capsules shell are the subject of great interest, as the modification in their micro-/nano-structures occurs when external light is used as a remote-control trigger [18,19,20,21,22,23].

Moreover, significant advantages emerge when light, over other external stimuli, is used to induce the release of the microcapsules core material: (1) the fine design of the photo-responsive molecular structure can precisely tune the excitation wavelength; (2) the time and/or local excitation are easy to control; (3) photons have very low or negligible toxicity and do not contaminate the reaction systems in contrast to chemicals (i.e., strong acids; oxidizing agents).

Various methods have been applied to develop such light-sensitive capsules with different functionalities [24,25,26]. One of the most common strategies for the synthesis of photo-responsive capsule shells, is the incorporation of molecular switches, such as azobenzene, stilbene, and other chromophores, into the polymeric shell structure. Azobenzene is one of the most recognized and investigated photochromic molecules. Pioneer studies concerning a functional optical behavior of azobenzene, based on reversible E-Z isomerization of the N=N bond upon photo-irradiation date back to 1930s [27]. Since then, this moiety has been extensively studied experimentally and theoretically [28,29]. The molecule is nearly planar in its thermally stable E conformation, while when irradiated with UV light (λ = 365 nm), produces the Z isomer, which has a bent conformation and a much larger dipole moment [30]. It has been reported that E-Z isomerization of azobenzene moieties could be caused by mechanical stress or electrostatic stimulation [31]. Furthermore, the process is reversible and thermal Z-E isomerization could occur spontaneously in the dark owing to the thermodynamic stability of the E isomer [32]. These molecular changes represent a powerful strategy for modulating microcapsule shell structural and functional properties in a controlled way. Various approaches have been performed to develop azobenzene-based photo-sensitive capsules with diverse functionalities [33,34,35,36]. As described by Woolley et al. [37], and later confirmed by our group [38,39], the modification of the azobenzene molecule with electron donating groups in the ortho position to the N=N bond shifts the E-Z photo-isomerization wavelength to visible light. In a previous paper [39] we reported the preparation of a modified azobenzene with methoxy group in ortho position and the use of this molecule for the preparation of visible light-responsive capsules. MC were prepared employing an oil-in-water (o/w) interfacial polymerization where the shell is formed at or across the surface of oil droplets by polycondensation of reactive monomers: oil-soluble ortho-substituted azobenzene monomer and water-soluble 1,8-diaminoctane and 1,3,5-Triazine-2,4,6-triamine (melamine) employed as a flexible spacer and cross-linker respectively. We demonstrated that the properties of the microcapsule walls and the release of encapsulated perfume oil could be triggered by exposure to visible light. 

In this work, we analyzed how the MC properties can be tailored by the precise design of the shell structure. The effect of three flexible spacers with different lengths (1,8-diaminooctane; 1,6-diaminohexane and 1,4-diaminobutane) and two types of cross-linkers (1,3,5-benzenetricarbonyl trichloride and melamine) on the release profile of the encapsulated perfume oil was investigated.

## 2. Materials and Methods

1,8-diaminooctane (DAO), 1,6-diaminohexane (DAH), 1,4-diaminobutane (DAB), 1,3,5-benzenetricarbonyl trichloride (BTC), 1,3,5-triazine-2,4,6-triamine, commercially known as melamine (MEL), Mowiol 18-88 (M18-88), xanthan gum, sodium hydrogenocarbonate, dimethyl sulfoxide (DMSO) were purchased from Sigma-Aldrich, Madrid, Spain and used without any further purification. 4,4’-bis(chlorocarbonyl)-2,2’-dimethoxy azobenzene (Azo) was synthesized by a previously reported procedure [39]. The perfume (P) with a composition described in the US granted patent No. 9890351 was provided by Procter and Gamble Services Company n.v., Brussels, Belgium. 

**Figure 1:**
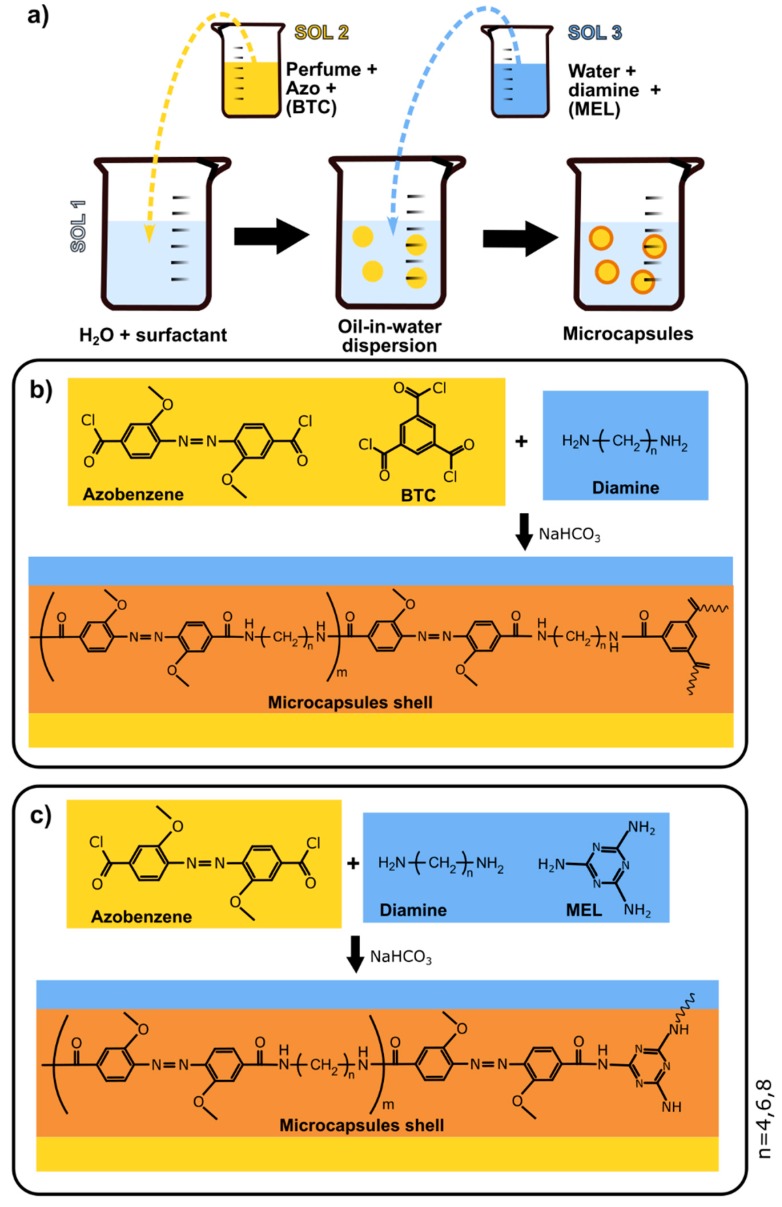
Schematic representation of (**a**) emulsion interfacial polycondensation and microcapsules shell formation using 1,3,5-benzenetricarbonyl trichloride (BTC) (**b**) and melamine (MEL) (**c**) as a cross-linker.

As mentioned above, MC were prepared by o/w interfacial polycondensation method, as reported in Figure 1a. MC1-MC3 capsules were prepared by using 1,3,5-benzenetricarbonyl trichloride (BTC) as the cross-linker (Figure 1b) while capsules MC4-MC6 were obtained with melamine as the cross-linker (Figure 1c). Due to the acid chloride moieties in the BTC structure and the amine groups in the MEL, theses cross-linkers were dissolved in aqueous and oil phases, respectively. Besides, three different flexible spacers: 1,8-diaminooctane (DAO), 1,6-diaminohexane (DAH), and 1,4-diaminobutane (DAB) were separately used for M1/M4, M2/M5 and M3/M6 capsules walls formation, respectively. Sodium bicarbonate was used to neutralize HCl formed during the capsule wall creation.

The amounts of each monomer used for MC shell formation are reported in Table 1. Following a standard protocol developed in our laboratory for polyamine capsules fabrication, three solutions were prepared separately:

- Solution 1 (Sol1)—50 mL of 1% M18-88 aqueous solution;
- Solution 2 (Sol2)—0.6 g (1.63 mmol) of azo and, for samples MC1-MC3, 0,067 g (0.25 mmol) of BTC were dissolved in 25 mL of perfume oil (P).
- Solution 3 (Sol3)—the listed quantities (see Table 1) of diamines (DA), 0.29 g (3.45 mmol) of sodium hydrogen carbonate and, for samples MC4–MC6 0.032 g (0.25 mmol) of MEL cross-linker were dissolved under gentle stirring in 25 mL of 1 wt. % M18-88 solution.

The o/w dispersion was obtained when Sol2, containing the acyl chloride monomer, was added dropwise to Sol1 and homogenized with an overhead stirrer at 1200 rpm for 20 min. Then Sol3, containing the diamine, was added dropwise into the dispersion to start the polycondensation reaction at the interface of the perfume oil droplet. The polycondensation reaction was performed at a stirring rate of 300 rpm for 3 h and stopped by dilution with 50 g of a water solution containing: 6 g of sodium sulfate, 0.350 g of xanthan gum and 43.6 g of demineralized water, to avoid the coalescence of microcapsules. The experiment was carried out at room temperature (25 ± 2°C) in a dark environment.

**Figure 2:**
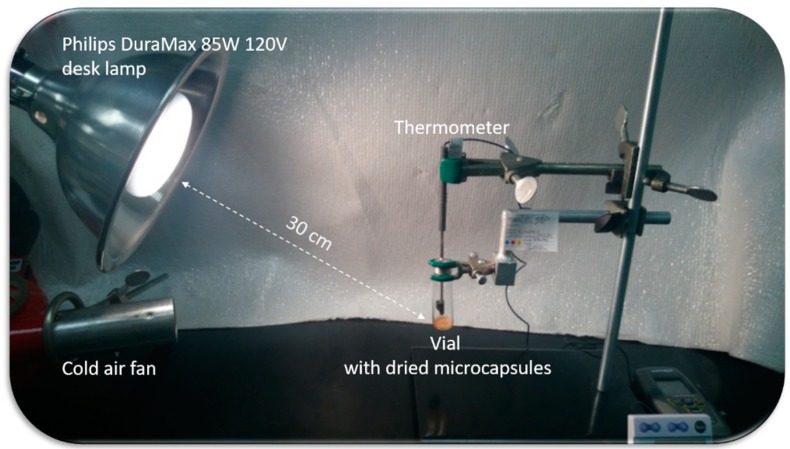
Release experiment setup.

The mean size of the polyamide microcapsule was determined by a Laser Diffraction Particle Size Analysis (LD) using a Helos BR supplied by Sympatec GmbH System Partikel Technik equipped with an R1 cuvette and a Helium-Neon Laser 5 mW max output at 633 nm (Sympatec GmbH, Clausthal-Zellerfeld, Germany). The analysis of 0.1 g of microcapsules slurry was carried out in 50 g of aqueous solution containing 6 g of sodium sulfate and 0.35 g of xanthan gum. Software setup and sample analysis were achieved using the Windox 5.8.0.0 software provided with the equipment by Sympatec, in Free Mode, using Fraunhofer Enhanced Evaluation. The data were collected twice, 5 s each. In order to verify the effect of visible light on the MC size, average capsules diameters were also measured after 3 h of samples irradiation with visible light emitted from a Philips DuraMax 85W 120V desk lamp. The distance between the light source and the quartz cuvette containing dispersed microcapsules was kept constant (approximately 30 cm). During the irradiation processes, cold air stream emitted from a cold air fan (25 ± 2 °C, monitored by means of a type-K thermocouple) was used to keep the capsule’s suspension in a thermostatic environment as shown in Figure 2. 

Scanning Electron Microscope (SEM) observations were performed at −70 °C in order to avoid chamber and detector contamination caused by vacuum-induced perfume release that may occur at room temperature. A Hitachi Model S-5200 Scanning Electron Microscopy (Hitachi High Technologies, Tokyo, Japan) equipped with a Gatan Alto 2500 Cryotransfer System (Gatan Model CT2500, Gatan, Inc. Pleasanton, CA, USA) was used. MC samples were immersed in liquid nitrogen at −210 °C, where the aqueous dispersion phase containing xanthan gum and sodium chloride is frozen forming the bulk mass. Liquid nitrogen fracture was performed to allow the observation of MC cross-section. Image-ProPlus 5 software was employed to analyze capsules dimension.

The photo-sensitive MC were observed by Nikon Eclipse E600 POL Optical Microscope (NIKON CORPORATION, Tokyo, Japan). During morphological observation, the instrument light was filtered with a Red 25 Kodak Wratten Color Filter in order to minimize MC morphology variations caused by the visible light emitted from the microscope bulb. On the other hand, a source of visible light (12V DC, 100 W halogen lamp Philips 7724, Philips North America LLC, Andover, MA, USA) was employed to evaluate light-induced modification in the MC shell. Capsules were irradiated for up to 25 min and then kept in darkness overnight.

UV-vis absorption spectra of ortho-substituted azobenzene solutions in DMSO were collected, before and after 2 min of irradiation with Philips bulb, by using a JASCO Mod. V570 spectrophotometer (JASCO International, Tokyo, Japan) with a double beam/single monochromator optical system.

To further elucidate the MC morphological changes under visible light at the nanometric scale, Atomic Force Microscopy analysis was used (AFM) [40]. The AFM studies were carried out by using a MultiMode AFM (Bruker, San Jose, CA, USA) equipped with air probe holder (MMEFCH or similar) and Silicon AFM probes for imaging in air, OMCL-AC160TS-W2 (Olympus, Tokyo, Japan) with the following nominal parameters: resonance frequency 300 kHz; spring constant 42 N/m; tip radius of curvature <10 nm, 7 nm. AFM data processing was performed using WSxM software, version 4.0 Develop 5.3 (Nanotec Electronica S.L., Madrid, Spain).

The BIOVIA Materials Studio program (Accelrys, San Diego, CA, USA) was used to create the bulk polymer in periodic boundary conditions starting off from the coordinates of the molecular models. The unit cell was then optimized by deploying the VAMP module using the Parametric Model 3 (PM3) semi-empirical method by Stewart, while 3-D models were created by using the periodic boundary conditions and an Amorphous cell module. Furthermore, molecular architectures of BTC and MEL were optimized by Molecular architectures BIOVIA’s toolset for engineering efforts.

Encapsulation efficiency (*EE*) is reported as the percentage of total encapsulated perfume in the MC slurries, derived from the amount of non-encapsulated perfume analyzed by applying a liquid–liquid extraction method with an n-octadecane as an internal standard, and gas chromatographic-mass spectrometric analysis (GC-MS) provided in the US Patent Application 9890351 and in Reference [39]. *EE* was calculated as follows:

(1) $$\%EE=\frac{A_{PE}\cdot RRF\cdot m_{ISE}\cdot 100}{A_{ISE}\cdot m_{E}}.$$

wherein: *m_ISE_* is the amount of n-octadecane in grams, *m_E_* is the amount of encapsulate’s composition (slurry) in grams, *A_PE_* is the area of perfume (sum of peaks) and *A_ISE_* the area of n-octadecane, *RRF* is the relative response factor calculated using the following equation:

(2) $$RRF=\frac{m_{P}\cdot A_{IS}}{A_{P}\cdot m_{IS}}.$$

wherein: *m_P_* is the amount of perfume is a standard solution, *m_IS_* is the amount in grams of n-octadecane, *A_IS_* is the area of the n-octadecane and *A_P_* is the area of the perfume (sum of peaks).

Perfume release was also determined using GC-MS and procedure reported in [39]. A measured amount (100 µL) of each MC slurry (MC1-MC6) was dried in darkness at 22 ± 2 °C and placed in a vial. Next, after 1 h of incubation in a jacketed reaction vessel at 22 ± 2 °C, the dried capsules were irradiated for 1, 2, 3, and 4 h, respectively, as previously described. The amount of released perfume was collected with a manual solid-phase micro-extraction (SPME) fiber (1 cm of SPME 75 mm Carbonex TM PDMS Fiber) for 20 s and measured by means of an Agilent 5975C Gas Chromatograph equipped with 5G4513A mass spectrometer (Agilent, Santa Clara, CA, USA). The GC-MS conditions were: 30 m HP-5MS column, initial temperature 45 °C, 1 min, 30 °C/minute, to 80 °C, then 8 °C/minute to 250 °C.

## 3. Results and Discussion

Six types of photo-sensitive microcapsules were synthesized by applying the interfacial polymerization method previously described. The encapsulation efficiency (EE) in samples MC1–MC6 is given in Table 2. Obtained results clearly show that the percentage of encapsulated perfume in these slurries is strongly influenced by the type of cross-linker used for microcapsules preparation. About 99.5% of perfume was encapsulated in the capsules prepared with the melamine as the cross-linker (samples MC4-MC6), independently of the type of diamine used for MC wall formation. On the other hand, in samples MC1–MC3, in which BTC was used as the cross-linker, the EE values ranged between 90% and 96% and depended on the length of the diamine employed. Indeed, it can be observed that a higher percentage of encapsulated perfume (EE = 96.0 ± 0.7) was achieved in MC3 where the shorter diamine, DAB, was used as the flexible space; on increasing the length of the flexible spacer, the amount of encapsulated perfume in the MC1-MC3 slurries slightly decreased. It should be taken into account that both families of slurries (MC1–MC3 and MC4–MC6) contain a rigid structured cross-linker; however, in the case of MEL, the azobenzene moiety is directly connected with the crosslinker, while in the case of BTC, a flexible diamine acts as a bridge. Furthermore, in the latter case, the shorter the amine, the better EE was obtained. The obtained results suggest, therefore, that a more rigid structure determines higher encapsulation efficiency. Actually, the highest EE resulted from the rigid MEL-Azo systems. 

Table 2 also reports the MC average size before and after 3 h of their irradiation with visible light. Interestingly, the MC size is not affected by the type of cross-linker or diamine used for microcapsule preparation but seems to be mainly determined by the overhead stirrer speed used for the o/w homogenization. In fact, MC1–MC6 mean capsules diameter, measured by LD, ranged between 62.5 and 68.7 µm. After 3 h of irradiation, the microcapsule size decreased approximately 16% proving that morphological changes are occurring in the shell structure.

**Figure 3:**
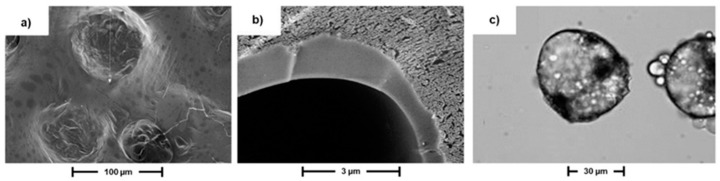
SEM analysis of MC3 surface (**a**) and cross-section (**b**) and optical micrographs showing MC3 internal morphology (**c**).

As an example of morphological characterization, Figure 3 shows the cryo-SEM micrographs of MC3 surface (Figure 3a) and cross-section (Figure 3b). According to SEM analysis, all obtained microcapsules appeared dispersed, well-formed and spherical, with a dense wall. On further observation, SEM micrographs reveal that some of the microcapsules are coated by an external layer. Indeed, this corresponds to the xanthan gum coating as well as to the crystallized sodium sulfate which were used to avoid capsules agglomeration in the slurries. Therefore, only an approximate shell thickness was measured during the SEM analysis of all slurries, which gave 49 ± 2 nm. In order to confirm the core-shell structure of prepared microcapsules, optical microscopy studies were employed. As Figure 3c shows, a difference between the particle surfaces and their borders was evident. 

**Figure 4:**
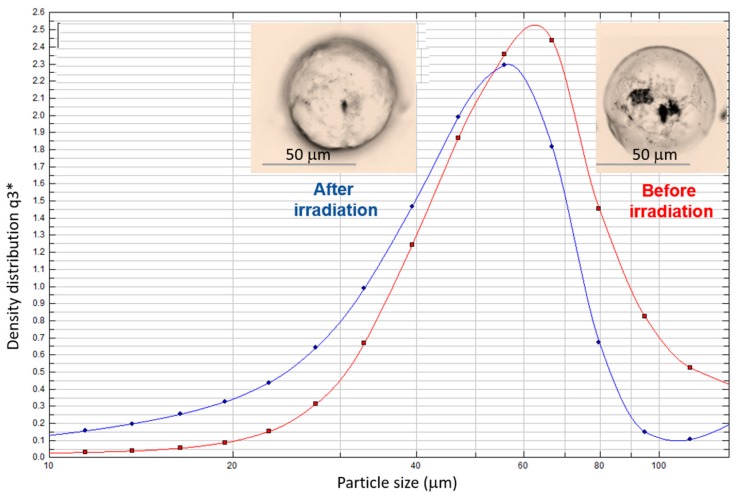
Optical microscopy of single MC4 microcapsule surface morphology changes and laser diffraction particle size analysis of MC4 microcapsules before and after 12 min of irradiation with visible light.

Optical microscopy was also used to investigate microcapsule surface changes during irradiation with visible light. Figure 4 shows a single microcapsule’s shape before and after its exposure to visible light emitted from the microscopy bulb for up to 12 min. Before the irradiation, the microcapsule seems to be well-formed with a smooth surface. Then, during the irradiation, it’s surface morphology started to change and appears rough. As a further example, LD studies on MC4 microcapsules slurry demonstrated that after 12 min of irradiation the average microcapsule diameters decrease by 16% from the initial. No further morphological changes were observed after 12 min to 3 h of additional irradiation. Interestingly, after additional 15 h of darkness, a recovery of the original microcapsule’s diameters was observed as well as the investigated single microcapsule surface has turned back to well defined and spherical, like at the beginning of the experiment. As a matter of fact, in our previous modelling studies performed on UV-sensitive microcapsules [41] and visible light triggered capsules [39], based on unmodified azobenzene and ortho-substituted azobenzene moieties, respectively, we concluded that the decrease of microcapsule size takes place due to E-Z isomerization upon corresponding light irradiation. On the other hand, the capability of the Z-isomer to relax back to its E form over time has been well demonstrated and discussed in the literature [42] and can justify the capsule size recovery observed in the current study. 

**Figure 5:**
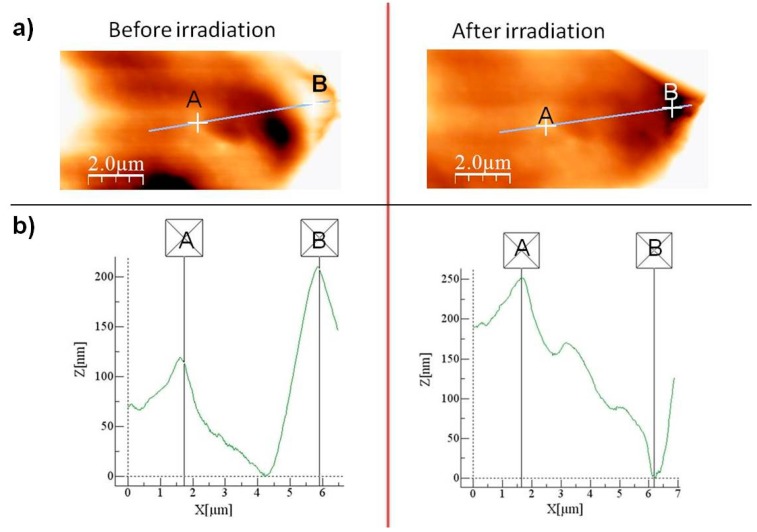
Atomic Force Microscope scan areas recorded in a tapping mode of sample MC3 before and after irradiation (**a**), AFM Z (height) between points A and B during irradiation of sample MC3 (**b**).

**Figure 6:**
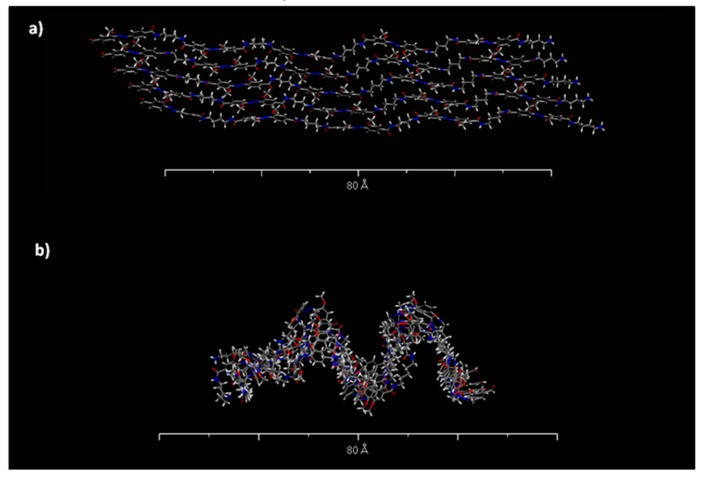
Topographies of polymer chains between neighboring cross-linker molecules, formed by azo and DAB units before (**a**) and after (**b**) irradiation.

**Figure 7:**
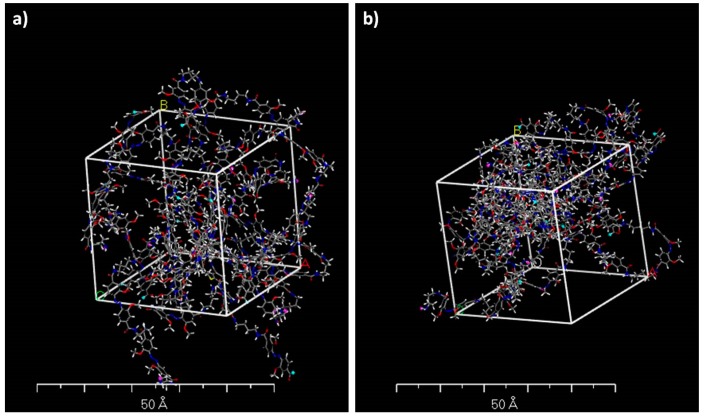
3-D structures of the simplified microcapsule formed with azo and DAB units between neighboring cross-linker molecules, before (**a**) and after (**b**) irradiation.

In order to follow the microcapsule’s surface topography changes induced by visible light, AFM studies were carried out and specific features in the AFM topographies were analyzed by line profiling routines provided in WSxM software. Figure 5a shows the AFM topographic micrographs of MC3 capsule before and after visible light irradiation. Linear scans (x-direction) and topographic analyses (z-direction) were performed between two labeled points A and B. As shown in Figure 5b, the microcapsule topography surface profiles between the analyzed points were changed after visible light irradiation that clearly indicates morphology modification due to the ortho-substituted azobenzene moiety photo-isomerization. To understand the mechanism of these changes, extensive computational studies were performed. After a successful 4,4’-bis(chlorocarbonyl)-2,2’-dimethoxy azobenzene conformational searching, geometry and energy optimization, the photo-sensitive polymer shell topographies were simulated. Materials studio software allowed building and visualization of surfaces formed by an uncross-linked polymer. We, therefore, wished to set up computational experiments whereby the changes in the geometric structure of polyamide chains incorporated between the cross-linkers can be inspected in the bulk polymer. For that purpose, we created 1-D models using periodic boundary conditions. Computational experiments were set up to monitor any changes in the polymer chains structure between neighboring cross-linker molecules. As a matter of example, Figure 6 shows the topographies of polymer chains formed by azo and DAB which simulate part of MC3 shell, while Figure 7 provides 3-D structures of the simplified microcapsule with azobenzene in E-form (a) and Z-form (b), respectively.

**Figure 8:**
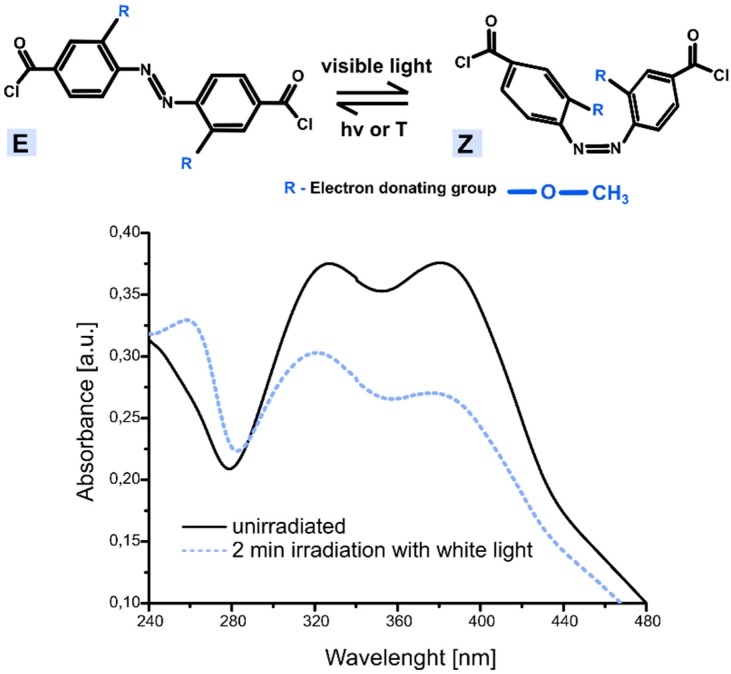
UV-VIS absorption spectra of ortho-substituted azobenzene at room temperature (**a**) before and (**b**) after 2 min irradiation with white light.

While the topographies containing the azobenzene polymers in E-form were greatly symmetrical (Figure 6a and Figure 7a) exhibiting planarity, the topographies formed by Z-form were much more geometrically irregular (Figure 6b and Figure 7b) and shorter per unit cell. As we measured from Figure 6, the Z-isomer polymers forming the shell occupied less space and were approximately 20 ± 3% shorter than the E-polymers, assuming total isomeric purity. These modeling studies provide a theoretical explanation for the ca. 16 % decrease of mean microcapsule size diameter after 3 h of irradiation with visible light (see Table 2). By comparing structure density before and after irradiation (Figure 7a,b), it seems that the irradiated microcapsule structures are much denser than prior to light exposure. This is due to the polymer length decrease caused by the E-Z isomerization of azobenzene which also could explain the AFM results. The E-Z photoisomerization of the ortho-substituted azobenzene moiety upon visible light irradiation, included in microcapsules shell, was confirmed by UV-Vis studies. Figure 8 provides the UV-Vis absorption spectra of ortho-substituted azobenzene molecule before and after illumination with white light. 

As it is shown in Figure 8, upon 2 min of light irradiation, the dominant π-π* band of ortho-substituted azobenzene became significantly less intense and the n-π* band is lightly blue-shifted and less intense. Moreover, upon irradiation, a new band appeared with high intensity at 257 nm [37]. 

The next step of the research focused on perfume release characterizations. As evidenced by Figure 8, the n-π* band was mostly centered on the blue-green light region, therefore it was expected that the irradiation with blue-green light results in a large fraction of the Z isomer, and thus in a more efficient release of the encapsulated core. However, our recently generated results on visible-light triggered nanocapsules based on the same ortho-substituted azobenzene moiety [43] put into evidence that a higher amount of released active was achieved upon visible/white light irradiation than within green light. For this reason, during current studies, visible light was used for perfume release investigations. 

**Figure 9:**
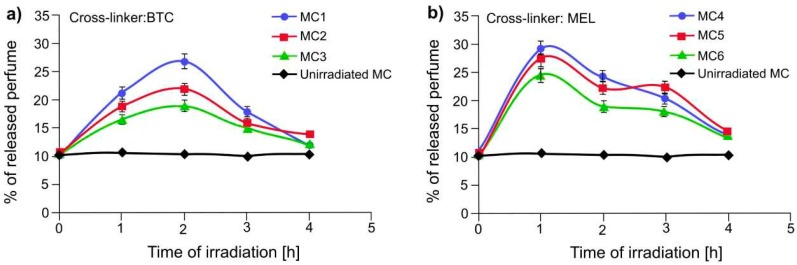
Percentage of released perfume from the polyamide microcapsules prepared with 1,3,5-benzenetricarbonyl trichloride (**a**) and melamine (**b**).

**Figure 10:**
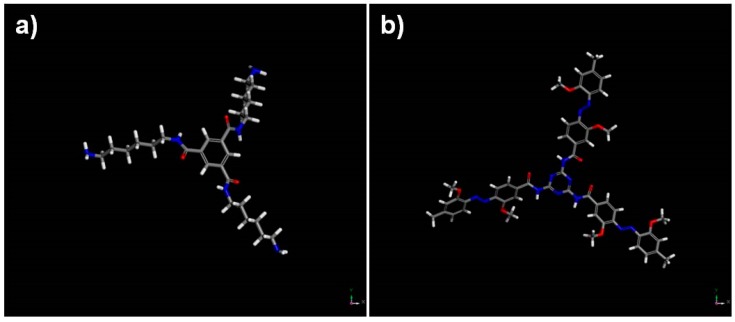
Molecular architectures of (**a**) BTC-DAE and (**b**) MEL-azo (after geometry optimization studies by DMol3 and Vamp modules) incorporated in microcapsule walls.

Figure 9a shows the percentage of released perfume to vial headspaces from the polyamide microcapsules synthesized using BTC as a cross-linker and DAO (MC1), DAH (MC2), DAB (MC3) as flexible spacers. Figure 9b provides the percentage of released perfume from the microcapsules formed with melamine as cross-linker and DAO (MC4), DAE (MC5) and DAB (MC6). At the beginning, 10% of perfume was already detected in vial headspaces. From our previous experience, this amount corresponds to perfume released as a consequence of the breakage of more brittle capsules during sample preparation. Figure 9 shows that about 18% of the total encapsulated perfume was released during the first hours of irradiation, then the amount of perfume available in the headspace decreases even upon continuous exposure. These results suggest that the released perfume either escaped the vials or was deposited onto the exterior of the vials. In order to determine the level of free perfume oil deposited on the vial internal surface, at the end of the perfume release experiment from MC4 microcapsules (after 4 h irradiation), 5 mL of hexane was injected to the vial and liquid-liquid extraction was performed, applying the same analytical protocol used for EE tests. The free oil, as measured by extraction, gave a value of 36.8 ± 0.5%. On the other hand, the cumulative amount of free oil released into the headspace, determined by taking the peak headspace value on each hour of measured release by the GC-MS/SPM protocol [39], gave 37.4 ± 0.7%. These two values overlap within the experimental error; therefore, it is reasonable to conclude that, after release, the oil partially deposited onto the vial wall. According to the literature, the mobility and stability of the azobenzene moieties in a polymer film are significantly dependent on the length and the rigidity of the spacers connecting them. On the other hand, it has been reported that when the flexible spacer is of sufficient length, such as 6–8 methylene units, spacer length does not meaningfully impact the alignment of azobenzene group [38]. Irie et al. [44] reported that if the azobenzene moieties are connected in the main chain of the polymer exclusively by flexible spacers, the azo conformational isomerization scarcely affects the shell’s overall conformation. According to the author, in that case, the flexible backbone acts to dissipate strain. Thus, the overall transformation of polymer shape does not occur because the conformational change encouraged by the photo-isomerization is relaxed in the connecting flexible chains. As a consequence, in the case of lightly crosslinked systems, as in our case, the structure and functionality of the crosslinker are expected to be the key factor for effective photo-release. Therefore, the more rigid the structure, the more effective light-triggering should be. Indeed, our results demonstrate that, during the first cycle of irradiation, the capsules containing melamine were able to release the encapsulated perfume faster than those containing BTC. To deepen this aspect for our systems, we performed modeling calculations of shear modulus (known also as modulus of rigidity, SM), which described a coefficient of elasticity for a shearing or torsion force of the molecular architectures. For instance, the calculated SM of BTC-DAE (Figure 10a) resulted in lower (SM 905 MPa) than MEL-Azo’s one (SM 1178 MPa, Figure 10b). This difference in rigidity of the two structures could explain why the encapsulated perfume is released 1 h later from MC1-MC3 systems than from MC4-MC6 on photo-irradiation.

**Figure 11:**
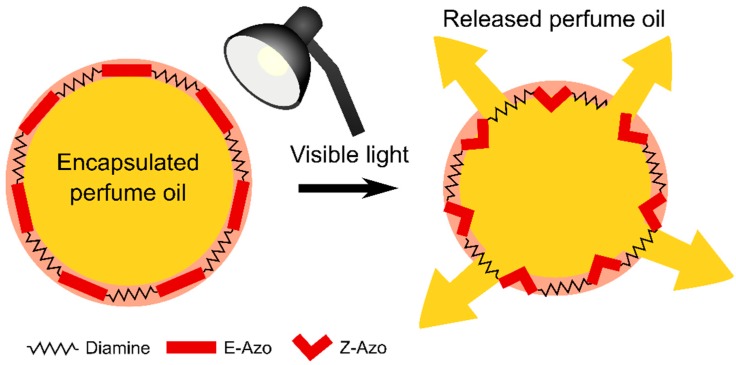
Schematic representation of release mechanism from azobenzene-based microcapsules.

Our previously published results [39,41] demonstrate that a release mechanism, from photo-triggered capsules based on azobenzene molecules incorporated in the main chain of polymer structure, occurs as a consequence of a squeezing effect due to E-Z photoisomerization, as shown in Figure 11.

## 4. Conclusions

In this work, photo-sensitive microcapsules, containing a perfume oil, were prepared by oil-in-water emulsion polycondensation of 4,4′-bis(chlorocarbonyl)-2,2′-dimethoxy azobenzene with three diamines with different length and two types of cross-linkers. Experimental studies showed that photo-isomerization of azobenzene moiety induced morphological changes and size decrease of the investigated microcapsules. Modelling studies confirmed that the release of encapsulated perfume not only depends on the incorporation of the photosensitive moieties in the capsule shell but also on the structure and functionality of the crosslinker used for the capsule fabrication. Actually, perfume release was faster in the systems prepared with MEL than BTC. These results suggest that it is possible to tailor the release profile of an encapsulated material by the molecular design of the capsule shell. Moreover, visible light is abundantly available, and it is not cancerogenic like UV light, therefore represents a promising source of energy for light-triggered release platforms in drug release therapy. 

## 5. Patents

List of patents related to the work: US9890351, EP2741731, JP5948417, MX-347260, RU2574030, CA2842348.

## Figures and Tables

**Table 1:** Monomers used for the preparation of polyamide microcapsules.

Sample	Sol 1		Sol 2			Sol3					
-	Waterc (mL)	M18-88 (g)	P (mL)	Azo (g)	BTC (g)	Water (mL)	M18-88 (g)	DA Type	DA (g)	MEL (g)	NaHCO_3_ (g)
MC1	50	0.5	25	0.6	0.067	25	0.25	DAO	0.25	-	0.29
MC2	50	0.5	25	0.6	0.067	25	0.25	DAE	0.20	-	0.29
MC3	50	0.5	25	0.6	0.067	25	0.25	DAB	0.15	-	0.29
MC4	50	0.5	25	0.6	-	25	0.25	DAO	0.15	0.032	0.29
MC5	50	0.5	25	0.6	-	25	0.25	DAE	0.12	0.032	0.29
MC6	50	0.5	25	0.6	-	25	0.25	DAB	0.09	0.032	0.29

**Table 2:** Encapsulation efficiency (EE) and microcapsules size before and after 3 h of irradiation with visible light determined by Laser Diffraction Particle Size Analysis.

Sample	EE (%)	MC Diameter (µm)	MC Diameter (µm) (after 3 h Irradiation with Visible Light)
MC1	90.8 ± 0.9	63.7 ± 2.0	52.7 ± 2.0
MC2	93.2 ± 0.9	62.5 ± 2.0	53.1 ± 2.0
MC3	96.0 ± 0.7	68.7 ± 2.0	59.8 ± 2.0
MC4	99.5 ± 0.2	63.3 ± 2.0	52.6 ± 2.0
MC5	99.5 ± 0.2	63.4 ± 2.0	51.9 ± 2.0
MC6	99.6 ± 0.2	62.6 ± 2.0	51.6 ± 2.0
